# Patients’ trust and associated factors among primary care institutions in China: a cross-sectional study

**DOI:** 10.1186/s12875-022-01709-8

**Published:** 2022-05-06

**Authors:** Liqing Li, Liyong Zhu, Xiaogang Zhou, Guohua Zeng, Hongwei Huang, Yong Gan, Zuxun Lu, Xiaofang Wang, Zhensheng Chen, Ke Sun, Di Yang, Qi Zhang, Chunmei Wu

**Affiliations:** 1 School of Economics and Management, Jiangxi Science and Technology Normal University, Jiangxi Nanchang, China; 2 School of Public Health, Tongji Medical College, Huazhong University of Science and Technology, Wuhan, Hubei China; 3 School of International Education, East China Jiaotong University, Nanchang, Jiangxi China; 4 School of Economics and Management, Jiangxi University of Science and Technology, Ganzhou, Jiangxi China; 5 Department of Health Management Medicine, The Second Affiliated Hospital of Nanchang University, Nanchang, China; 6 School of Public Health and Health Management, Gannan Medical University, Ganzhou, Jiangxi China

**Keywords:** Patients, Trust, Influencing factors, Primary care institutions

## Abstract

**Background:**

Empirical evidence on patients’ trust and the factors among primary care institutions (PCIs) in China is limited. This study aimed to investigate patients’ trust and explore some associated factors among PCIs in the central region of China.

**Methods:**

The data was collected through a multistage stratified sampling method with a structured self-administered questionnaire, which was distributed from January to March 2021 among 2,287 Chinese patients ever involved in seeking healthcare among PCIs. Patients’ trust was measured with the Chinese version of the Wake Forest Physician Trust Scale (C-WFPTS). Differences in C-WFPTS scores among groups were estimated by *t*-tests or ANOVA analyses. Multiple linear regression analysis was used to analyze influencing factors for patients’ trust in primary care physicians.

**Results:**

Based on the C-WFPTS with a full score of 50, the average score of patients’ trust was 34.19 (SD = 5.83). Multiple linear analyses indicated that the patients who were older aged, married, with education of higher level, living in urban regions, under better health status and with a family doctor contract reported a higher level of patients’ trust.

**Conclusion:**

Patients’ trust in primary care physicians was at a medium but slightly improved level in the central region of China. Age, marital status, education, residential area, health status, and a family doctor contract were significant predictors of patients’ trust.

## Background

Primary care is the backbone of a nation’s health care system [1]. The institute of Medicine in America has defined primary care as the necessary provision of integrated and accessible health care services with clinicians, who are accountable for a large majority of personal health care needs, developing a sustained partnership with patients in practice of the context of family and community [2]. China’s medical institutions mainly include hospitals and primary care institutions (PCIs) [3]. Hospitals are divided into three levels according to the numbers of beds and their respective functions. PCIs include community health service centers or stations in urban regions, township health centers and village health posts in rural areas, as well as school infirmary and private clinics [4]. In China, hospitals and PCIs have delivered the majority of medical care services [5]. In 2020, 42.73% of outpatient care and 79.75% of inpatient care were in hospitals, while 53.00% of outpatient care (4.12 billion visits) and 16.11% of inpatient care (37.07 million hospital admissions) were provided by PCIs; Among these, 68.69% (2.83 billion) of outpatient visits and 98.60% (36.55 million) of the hospitalizations were from government-sponsored PCIs [6]. PCIs provide generalist clinical care and basic public health services, which have contributed greatly to reductions in the burden of diseases in China.

Compared with hospitals, PCIs are much less capable to attract health resources, workforce and technology. For instance, the average number of licensed doctors in one community health center, community health station, township health center and village post was 18.2, 2.0, 1.5, and 0.8 licensed doctors on average in one community health center, community health station, township center and village post, respectively [6]. By contrast, each hospital had 81 licensed doctors averagely, totally 70% of all the licensed doctors in the country [6]. There were still a certain amount of unlicensed primary care doctors, especially in rural PCIs [7]. Multidisciplinary professional teams are required in PCIs to be responsible for primary care and basic public health needs to a certain population, but the workforce are not evenly distributed, and many doctors are either public health physicians or specialists rather than general practitioners [7, 8]. In 2016, the family doctor contract services (FDCS) system was launched in China as an important project of China’s new health care reform to improve the quality of primary care [9]. After signing a contract with a family doctor team in a PCI on a voluntary basis, the resident could have a higher reimbursement rate, and/or preferential or even free access to certain primary health care, like health management and follow-ups varying from province and municipal. However, the worries remained about the quality of health services and the preferences for FDCS and PCIs had been not improved largely [10, 11] as the health care systems in most municipals do not restrain residents to choose medical facilities much, even those with contracts for FDCS.

For a long time unfortunately, there existed a serious resource shortage in China’s PCIs, which finally caused the distrust from consumers or patients. Services delivered by PCIs, considered as being poor quality, were unable to function well as a gate-keeper in a health care system. It was common for patients to go first to a hospital just for a minor illness or a general chronic condition. However, primary care can also play a crucial role in promoting regional health equity via its large number of widely distributed institutions of better accessibility [12]. In addition, due to the high health demands but low trust in PCIs [13], patients would definitely go to hospitals first rather than PCIs when they were sick [14].

Trust is a major drive force for human relationships of all kinds. Trust in healthcare field has been receiving increasing attentions for decades of years [15]. Studies have shown that trust in doctors is associated with positive health behaviors of patients [16]. It is a forward-looking covenant between doctors and patients within which patients believe doctors would behave for their best interests [17]. It is also defined by patients as an optimistic belief or acceptance of vulnerability that doctors would conduct treatments with their moral character and competences [18]. Empirical studies have revealed that patient trust in doctors is associated with patient satisfaction [19], continuity of care [20] and adherence to treatment [21]. Trust in doctor facilitates an access to healthcare, disclosure of relevant information and thereby supports a doctor in making an accurate diagnosis in time [22]. The degree of patient’s trust in doctors has reflected the important implications for treatment outcomes [23]. Higher trust was associated with patients’ greater care-seeking behaviors, greater adherence to treatment regimens and better involvement in treatment decision-making [24]. Less trust would result in patients’ low adherence to treatment plans, increased demands for referrals and diagnostic tests, and ultimately poor health outcomes [23]. Trust is a multidimensional concept and several validated tools have been developed to evaluate patients’ trust in doctors, including the Trust in Physicians Scale [25], the Primary Care Assessment Survey [19], the Patient Trust Scale [26], and the Wake Forest Physician Trust Scale (WFPTS) [20]. However, it is still in an initial stage for developing scales for patients’ trust in China, and the adaptation of the Chinese version of the Wake Forest Physician Trust Scale (C-WFPTS) is one of the most widely used scale [27–31]. The C-WFPTS shows good psychometric properties in evaluation of patients’ trust in patient-doctor relationship, and therefore it provides an essential tool for the characterization of patient-doctor relationship in China [32].

Although most patients would still like to trust that doctors would conduct medical treatments for patients’ best interests, the declining trust scale still attracted growing concerns [33–35]. Some previous studies have investigated patients’ trust among hospitals and some influencing factors, such as patients’ gender [27], age [27], education experience [36], occupation, and income level [23]. However, empirical evidence on the patients’ trust in doctors and its determinants is limited in Chinese PCIs. Therefore, this study aimed to contribute to the limited literature on patients’ trust in primary care providers by using a validated measure. This study also added to the empirical literature on the influencing factors for trust, most of which were social and demographic features, which provides evidence for the identifation of people with less trust and making individual measurements to improve trust and patients' care seeking decisions in China.

## Methods

### Study population

This was a cross-sectional study conducted among the PCIs in central region of China from January to March 2021. A multistage stratified sampling survey was carried out. In the first stage, 5 provinces out of six provinces in central China were randomly chosen, i.e. Jiangxi, Hunan, Hubei, Anhui, and Henan. In the second stage, 5 township health centers in administrative villages and 5 community health service centers or stations were randomly selected from 3 cities in each province. In the final stage, patients were selected on convenience and the number in each PCI depends on the scales of the selected PCIs.

The median number of beds for community health centers was fourty [7]; thus, we investigated 40 patients in a PCI with more than 40 beds, and 20 in a PCI with less than 40 beds. Eligible criteria were: (1) aged 15 years or older, (2) be healthy and educated enough to fill the questionnaire, (3) be willing to participate. Respondents were asked to complete a self-reported questionnaire via WeChat to avoid contact considering the COVID-19 epidemic. WeChat is one of the most popular mobile messaging applications in China with hundreds of millions of active accounts. To improve the completion rate, respondents could not submit the questionnaire before answering all the questions. Finally, a total of 2,287 out of 2,300 questionnaires were collected with a response rate of 99.43%.

All participants provided written informed consents before their participation in the study. The study protocol was approved by the institutional review boards of Tongji Medical College, Huazhong University of Science and Technology, Wuhan, China.

### Instrument and measurement

A self-reported questionnaire was designed. It contained an introduction and informed consent (Part I), collected basis information on the sociodemographic factors, health status and family doctor contract of the individual and his/her household (Part II) as well as trust in PCIs using the C-WFPTS (Part III). The sociodemographic characteristics included gender, age, marital status, residential region, education level, occupation, annual household income level, and number of family members. Self-evaluated health level and history of chronic diseases were asked to evaluate the health status of the respondents. Marital status was divided into five groups: married, unmarried/single, divorced, widowed, and other status. Education level included junior secondary school and below, senior secondary school (including secondary technical school), junior college or university, and bachelor or above. Respondents were asked to assess their annual household income level into six categories: less than 50, 50–100, 100–200, 200–300, 300–600 and over 600 thousand yuan per year. Physical health was recorded with a 5-point Likert scale (“1 = very poor; 2 = poor; 3 = general; 4 = good; and 5 = very good”). The 10 items from the C-WFPTS based on a five-point Likert scale, ranging from 1 (total inconformity, i.e. total distrust) to 5 (total conformity, i.e. total trust). The total scores ranged from 10 to 50, with higher scores indicating a stronger patients’ trust in PCIs.

A Cronbach’s alpha test was calculated to measure the internal consistency of the 10 Likert items to see the reliability when assessing patients’ trust in this study. The Cronbach’s alpha coefficient for the scale was 0.717, which indicated a good internal consistency.

The validity of the C-WFPTS in this study was also tested. The scale Kaiser–Meyer–Olkin (KMO) with 0.899 and Bartlett test (*χ*^2^ = 8,448.344, *P* < 0.001) collectively showed that the questionnaire was applicable.

### Data collection and quality control

The questionnaire was designed on the basis of literature reviews, group discussions, and mock interviews with a pre-test carried in PCIs in Nanchang, Jiangxi Province to improve the quality of the questionnaire. The web-link version of the questionnaire was made on a specialized free online questionnaire service website called Questionnaire Star (wjx.cn) and then disseminated online to patients through WeChat. The data was automatically collected by Questionnaire Star and stored into the Web-based database and then downloaded by the investigators.

### Statistical analysis

As a dependent variable in the study, patients’ trust in doctors was treated as continuous variable. In the multivariable linear regression model, predictive variables included gender, age, residential region, marital status, occupation, education level, annual family income level, chronic diseases, number of family members, health status, and the contract with family doctors. Multicollinearity was assessed with variance inflation factors [37].

Some covariates were recategorized to be more comparable to the national population. The “bachelor degree and above” education level was added with the “junior college and university” level. The final cut-offs of the family income levels were ¥50,000 and ¥100,000 per year. Some small groups merged if unevenly distributed. Then there were two groups by marital status (married and others), and three levels of health status (“1 = poor; 2 = moderate; 3 = good”).

IBM SPSS version 21.0 was employed for database assembling and statistical analysis. Descriptive statistics for distribution were calculated, and *t*-tests and ANOVA were used to compare the differences between means. Multiple linear regression analysis was applied to explore factors affecting the scores of patients’ trust in general practitioners in PCIs. Independent variables were included in the model if they significantly contributed to the prediction of total patients’ trust scores. The standardized coefficient Beta was used to distinct the effect size of each factor. *P* < 0.05 (two-tailed) was considered statistically significant.

## Results

Basing on the full score 50 of C-WFPTS questions, the average score of patients’ trust was 34.19 (SD = 5.83) and the median was 34.00. Table 1 reports main characteristics of the respondents from PCIs’ patients. There were a total of 2, 287 participants aged from 18 to 76 years (M = 32.16, SD = 12.016). Among them were more than a half (55.49%) female, more than a half (53.52%) married, more than two thirds (68.39%) having a college degree or above, more than a half (59.47%) freelancers, only 411 (17.97%) enterprise employees and 367 (16.05%) from public sectors. There were about one quarter (26.41%) from rural and more than two thirds (69.17%) with more than three family members. In total, there were more than six tenths (64.01%) with annual family income moderate or low, a majority (79.45%) without family doctors contracts, and a minority under poor health conditions (9.36%) or with at least one chronic disease (12.46%).

**Table 1:** Characteristics of respondents and their trust among PCIs (*n* = 2,278)

Variables		Frequency (%)	M ± SD	*t/F*	*P*
Sex	Male	1018(44.51)	33.88 ± 5.60	-2.278	0.023
Marital status	Female	1269(55.49)	34.44 ± 5.98		
	Married	1224(53.52)	34.91 ± 6.09	-6.462	< 0.001
	Unmarried/widowed/divorced/other	1063(46.48)	33.36 ± 5.39		
Education level	Junior secondary school or below	341(14.91)	33.86 ± 5.76	1.164	0.312
	Senior secondary school (including secondary technical school)	382(16.70)	33.97 ± 5.67		
	University/junior college or above	1564(68.39)	34.32 ± 5.87		
Occupation	Peasant	149(6.52)	33.81 ± 5.66	7.532	< 0.001
	Enterprise staffs	411(17.97)	33.82 ± 5.57		
	Personnel in government offices or public institutions	367(16.05)	35.50 ± 5.90		
	Freelancer	1360(59.47)	33.99 ± 5.86		
Area of residence	Urban	1428(62.44)	35.00 ± 6.10	41.362	< 0.001
	Rural–urban continuum	255(11.15)	33.68 ± 5.49		
	Rural	604(26.41)	32.50 ± 4.85		
Number of family members	Two or less	81(3.54)	34.52 ± 6.21	4.074	0.017
	Three	624(27.28)	34.73 ± 5.94		
	Four or more	1582(69.17)	33.96 ± 5.75		
Family income level (Yuan per year)	Low (< 50,000)	598(26.14)	33.71 ± 5.98	3.365	0.035
	Moderate (50,000—100,000)	866(37.87)	34.51 ± 5.75		
	High (> 100,000)	823(35.99)	34.20 ± 5.78		
Chronic disease history	Yes	285(12.46)	33.19 ± 5.55	-3.106	0.002
	No	2002(87.54)	34.33 ± 5.85		
Self-reported health status	Poor	214(9.36)	31.07 ± 5.07	58.084	< 0.001
	Moderate	676(29.56)	33.31 ± 5.7		
	Good	1397(61.08)	35.10 ± 5.76		
Whether having a contract with family doctors	Yes	470(20.55)	35.97 ± 5.81	-7.528	< 0.001
	No	1817(79.45)	33.73 ± 5.74		
Age	-	32.16 ± 12.01	-	-	-

*PCI* Primary Care Institution, *M* mean, *SD* standard deviation

Table 1 shows *t*-test and ANOVA analysis results, comparing the difference in mean scores of patients’ trust among groups. There were significant differences in patients’ trust in terms of gender, occupation, marital status, education level, residence region, number of family member, chronic disease history, family doctor contract, and health status (*P* < 0.05). The average scores of the trust scale varied among patients on different annual family income levels, but the differences were not significant (*P* = 0.394).

Table 2 shows the results from the multiple linear regression analysis, identifying factors associated with patients’ trust in doctors from PCIs. The indicated factors significantly associated with the patients’ trust were gender, age, marital status, education level, residence region, health status, and family doctor contract. And the higher patients’ trust were from those: female, older, married, better educated, living in urban regions, having better self-reported health, and with a contract with family doctor. There was no multicollinearity in this study because the variance inflation factor of each of the above variables was below the cut-off value of 10.

**Table 2:** Multivariable linear model for the association with the patients’ trust among PCIs*

Variables (reference)	*β*	SE	Standardized Coefficients beta	*t*	*P*
Sex (Female)	-0.424	0.234	-0.036	-1.812	0.070
Marital status (unmarried/widowed/divorced/other)	1.305	0.341	0.112	3.823	< 0.001
Education level	0.451	0.194	0.057	2.326	0.023
Area of residence (Rural)	1.450	0.252	0.121	5.760	< 0.001
Family income level	-0.036	0.154	-0.005	-0.235	0.814
Health status	1.832	0.190	0.208	9.656	< 0.001
Chronic disease history (No)	-0.450	0.377	-0.025	-1.191	0.234
With a family doctor contract (No)	2.026	0.294	0.141	6.901	< 0.001
Age	0.036	0.015	2.431	2.431	0.015
Constant	25.443	1.134	-	22.445	< 0.001

*PCI* Primary Care Institution, *SE* standard error

*R*^2^ = 0.121; *F* = 22.305, *P* < 0.0001

^*^ Adjustment for race (Han race or other race), occupation (farmer, enterprise staff, civil servants in government or public institutions, others) and number of family members (two or less, three, three or more) in the model

## Discussion

Primary health service demands among community residents are increasing in China. To turn residents’ choices of first contact care from hospital into PCIs, Chinese government has taken various measures to push for a reform of the medical and health care system. To meet people’s health demands, China has gradually established a hierarchical diagnosis and treatment system, which classifies diseases in accordance with the severity and urgency, enabling medical institutions of different levels to handle diseases of different types and stages according to various institutions’ functions. Although initial diagnoses in PCIs with a two-way referral system have been improving medical treatment order in China, the expected effects were not observed in the health care reform [3, 29]. It is important to improve patients’ trust in PCIs for the provision of safe and high-quality primary medical care. In addition, it strengthens the quality of primary medical care by identifying determined factors associated with patients’ trust.

This study investigated the patients’ trust in primary care doctors and relevant determinants in China. With a mean score of 3.42 and a median score of 3.40 for each question of the C-WFPTS, it found that Chinese people’s trust in primary care doctors would be slightly improved in recent years, but still relative low compared with many countries in the world [32, 38, 39]. It was difficult to compare the level of trust as in the absence of a gold standard for measuring trust. Generally, trust of Chinese people would be “medium” between “trust” and “distrust”, according to either “the average trust score” [32] or the proportions of different levels of trust [38, 40]. That may rely on the launching of the general practitioner system and a village doctor training program across the country since 2011 [41, 42]. China's fight against COVID-19 could also have contributed to the improvement [43, 44]. When comparing with people from other areas in the world, the public in China always showed high distrust in health-care settings, especially in primary health-care providers [38]. A large nationwide investigation was carried out with the same scale measuring patients’ trust as used in 28 countries across the world [40]. The results showed that only 25.2% Chinese residents trusting in medical services, and if there was no high level of public trust in the local government, the positive impact of our public trust in the health system would be even less optimistic. Being a part of the doctor-patient interaction, trust was highly related to one’s medical experience. As trust in government has always been high, and individuals’ social and demographic features could hardly be changed, changing public perception with the experience of health care is the primary, if not the only, way to increase public trust in the health care system [38, 40]. Calls should be made for a more patient-centered health care system, especially in primary care system, and it is critical to measure and understand patient perceptions of health care quality.

The associations between respondents’ trust in general practitioners and sociodemographic factors as well as health utilization were estimated. There were significant correlations of patients’ trust with age, marital status, education level, residence region, self-reported health status and family doctor contract. No significant relationship was observed between trust with chronic disease history and family income level, which were similar to previous studies [40, 45]

Age was found to be positively related to patients’ trust in previous studies [30, 32, 46–49]. In this study, there was also a statistically significant association between patients’ trust and age, which indicated that the youth were of lower patients’ trust in doctors among PCIs. This is comparable to other observations in China [23, 32, 38–40]. From the aspect of age, the adults older often keep more frequent contact with doctors and are much more dependent on doctors. Thus, the older are considered to have a higher trust for their doctors [27]. But distrust in PCIs could also arose with greater hospital utilization [38]. In addition, senior patients are more likely to be suffered from chronic diseases (for example, 52.8% of the respondents aged ≥ 65 years and 11.8% of those aged < 65 years were with chronic diseases in this study) and under poor health conditions, which could lead to lower health expectations and higher patient satisfaction, medical adherence and continuing enrollment, which all help them keep a higher trust in doctors. In our study, the association existed even after adjusting for chronic disease history and self-reported health status. Still some evidence showed opposite results [50].

In addition, the study showed that the patients with higher education had higher trust than those with less education, which might be explained by the fact that the community residents with a higher level of education may have a better occupation. They would be easier to access to health services and hence to decide the health service providers [36]. Another explanation could be that better educated people could express their expectations in the doctor-patient relationship more clearly than those with lower education [36]. Negative or ambiguous associations between trust in primary care physicians and their education level were found in other observations in China [23, 32, 38–40]. Intriguingly, education was significantly associated with patients’ trust in western counties [48, 51, 52], which was consistent with our study. The relationship between education and trust remains complicated.

The study found that residents’ trust in physicians was positively associated with the self-reported health condition and FDCS. The standardized coefficients showed that the influences of these two factors were the largest. It is well-known that primary care doctors in PCIs play a less important role than specialists in hospitals in China and those in other countries as well [23, 53]. Chinese residents, especially those with poor health status, have very few trust in competences of primary care providers; thus, they would seek health-care resources directly from hospitals once they get sick. Individuals with family doctor contracts have better access to and spend more time with preferred general physicians, which improve their perceived primary care quality and benefits in satisfaction and trust in PCIs [54]. The signing coverage rate in China of family doctor’s contract is still very low after the FDCS was launched in 2016. Wu et al. found that less than 1/4 (320 million residents) had under FDCS until 2018 [55]. Previous studies have shown that FDCS could bring more trust, and residents’ awareness and willingness should be encouraged for a higher coverage rate of FDCS [10, 11, 54]. Meanwhile, family doctor’s services should cover key services to meet local people’s health needs, and a performance assessment should be conducted so that the quality of contact services would not be neglected.

The study showed that female residents had a little higher level of trust than male residents (34.44 vs. 33.88), which was comparable to some observations in previous studies [29, 56]. In contrast to men, women were characterized by a desire to establish and maintain relationships with the particular relevance to the trust domain [57, 58]. Therefore, females are usuanlly more likely to trust their doctors. But the difference of trust between males and females was not significant after adjusting for other variables, implying more covariates to control related to patients’ experience of using primary health care.

### Strengths and limitations

There were several strengths in the study. Firstly, this study provided novel data on patients’ trust in primary care doctors and its influencing factors in China at the end of its first stage of establishing a general practitioner system and a village doctor training program as well as the outbreak of COVID-19 in 2020. Secondly, it had a large sample size and got a high response rate. The large sample size significantly increased statistical power to detect social determinates of respondents who sought health care from PCIs. Thirdly, it adopted a web-based survey approach provided by the online questionnaire service website Questionnaire Star (wjx.cn) and through the communications application WeChat, which was accessible for over 1 billion users with the popularity of smart phones and rapid development of communication tools in China. Due to the free platforms, it could minimize missing data as the respondents could not submit the questionnaire until covering the required data field even when they were unable or unwilling to complete the whole questionnaire.

However, some limitations should be acknowledged in the study. As the study focused on sociodemographic characteristics and only a few of health and medical experience factors were analyzed, it might be a failure to identify all potential factors influencing patients’ trust. In addition, a cross-sectional study design in this study precluded evaluation of the temporality and causality in the observed relationships. Finally, as selection bias was brought by the sampling pattern and the “mandatory” questionnaire, the results should be interpreted carefully. Compared with the 2020 national population [59], our sample were similar in the urban–rural-ratio, but younger, with a smaller sex ratio and much better educated.

The low level of trust in PCIs and primary care doctors is still a prominent issue in China after decades of healthcare reform measures by the government. The findings from this study indicated a possible improvement of trust for young, less-educated, relative unhealthier residents living in the community. Research is required on the underlying reasons why they trust less than others in primary care professionals, and to understand more fully what drives the distrust. While individual measures should be taken aiming at those who trust less, probably by improving patients’ perceptions of quality in health care.

### Conclusions

In summary, patients have a medium trust in their doctors when they seek healthcare from PCIs in China, and trust a little more than they used to. Some factors are significantly associated with the patients’ trust, such as age, marital status, education level, residence region, health status, and family doctor contract. Patient-centered health care system is needed and measures should be taken by China’s government to improve the patients’ trust in doctors for developing the primary healthcare mainly by improve their perceived quality of health care in young less-educated residents with poor health.

## Data Availability

The datasets used and/or analyzed during the current study are available from the corresponding author on reasonable request.

## Abbreviations

PCI: Primary care institution
C-WFPTS: Chinese Wake Forest Physician Trust Scale
FDCS: Family doctor contract services
WFPTS: Wake Forest Physician Trust Scale
KMO: Kaiser-Meyer-Olkin
